# Examining the quality of the competency-based evaluation program for dentistry based on the CIPP model: A mixed-method study

**DOI:** 10.34172/joddd.2021.034

**Published:** 2021-08-25

**Authors:** Nemat Bilan, Ramin Negahdari, Hakimeh Hazrati, Saeid Foroughi Moghaddam

**Affiliations:** ^1^Department of Medical Education, Medical Education Research Center, Health Management and Safety Promotion Research Institute, Tabriz University of Medical Sciences, Tabriz, Iran; ^2^Department of Medical Education, Center for Educational Research in Medical Sciences (School of Medicine, Iran University of Medical Sciences, Tehran, Iran

**Keywords:** Dentistry, Evaluation, CIPP model, Program evaluation

## Abstract

**Background.** Continuing assessment of the quality of evaluation programs promotes the quality of exams and ensures learners’ accurate evaluation. This study aimed to examine the quality of the competency-based evaluation program for dentistry based on the context, input, process, and product (CIPP) model.

**Methods.** In a mixed-methods study (quantitative-qualitative), dentistry students’ evaluation program using competency-based assessment methods was examined by dentistry professors based on the CIPP model and via a reliable and valid researcher-made questionnaire. The questionnaire had three sections on demographic information, evaluation questions, and open-ended questions. Descriptive statistics (mean and SD) were used to analyze the questionnaire items. Open-ended questions were analyzed by content analysis, and the quantitative part was analyzed using SPSS.

**Results.** Twenty-five faculty members from the departments of orthodontics and dental prosthesis completed the questionnaires. The overall level of faculty members’ satisfaction with the new evaluation program was above average (54±17.02). They had the highest degree of satisfaction with output indices and the lowest degree with input indices. The analysis of the open-ended questions yielded two general categories of "providing the human and physical infrastructure" and "spiritual support and encouragement of educational innovation."

**Conclusion.** The competency-based evaluation program needs the support of managers and planners. The faculty should provide the infrastructure for the implementation of these methods. By meeting the requirements, the professors will be motivated to implement these methods, and the paradigm can shift from traditional to novel evaluation methods.

## Introduction

Continuing program evaluation and identifying its strong and weak points are essential to having an efficient educational system.^1^ Educational program evaluation is an activity aiming to determine the quality and value of educational programs or processes, with the primary goal of making a value judgment about the implemented programs and presenting a model for educational planners and policy-makers to modify and, therefore, promote the quality of programs.^2^ The evaluation is valid when it is performed based on clear and comprehensive indices.^3^ The context, input, process, and product (CIPP) evaluation model is a management-based evaluation model that enables program evaluation during and after implementation in four dimensions of context, input, process, and output. In the dimension of context, the needs, goals, and conditions of achieving the program outputs are evaluated.^4^ In the dimension of input, all the factors affecting the achievement of educational goals, such as human resources, financial sources, strategies, and the program execution timetable, are evaluated.^4^ With this dimension, one can examine various solutions to realize educational goals and prevent using unnecessary facilities for achieving these goals.^5^ In the dimension of process, the program execution flow in the real world and its compatibility with the pre-determined goals and timetable are evaluated. Finally, in the dimension of output, the effectiveness and efficiency of the program in achieving the pre-determined goals are evaluated.^4^ The results of the evaluation provide authorities and decision-makers with information about continuing or aborting the program.^6^ In various departments of dentistry, educational programs have been evaluated based on the CIPP model. For instance, in a study by Tabari, the realization of educational goals was evaluated in three departments of operative dentistry, pediatric dentistry, and orthodontics. Based on this model, Babol University of Medical Sciences had realized its educational goals at an optimal level.^7^ According to Pakdaman et al, the realization of educational goals based on CIPP in the periodontics and oral health department requires revision in two input and process dimensions.^8^ In the study by Makarem et al in Mashhad Dental School, the educational program of the department of oral health needs revisions in the domain of process based on the CIPP model.^9^ Based on the evaluation of the dentistry program at the University of Birmingham, the dentistry students had poor capabilities in terms of root canal treatment and were not quite familiar with the equipment and techniques of this discipline.^10^ Henzi et al^11^ in the United States also evaluated dentistry educational programs from the learners’ viewpoint in some departments and identified the problematic areas. Student evaluation is an integral part of educational programs. In the Faculty of Dentistry, Tabriz University of Medical Sciences (Iran), modifications have been made to the dentistry evaluation program in line with a reform plan, and competency-based assessment methods have replaced traditional methods that relied on multiple-choice tests and professors’ opinions. The review of the literature showed that the evaluation program of dentistry using a competency-based approach has not been evaluated yet. Therefore, this study aimed to assess the dentistry competency-based evaluation program based on the comprehensive CIPP model to identify the strong and weak points of the program and provide insights for planners to promote the quality of exams.

## Methods

The data in the present mixed-methods (quantitative-qualitative) study were collected via a reliable and valid researcher-made questionnaire based on the CIPP model and open-ended questions, with the professors’ voluntary participation Departments of Orthodontics and Prosthodontics, Faculty of Dentistry, Tabriz University of Medical Sciences.

This study was conducted in three stages.

Stage 1 consisted of designing and extracting the evaluation questions of the “implementation of professional competency-based evaluation program based on the CIPP model” in four dimensions of context, input, process, and output. Stage 2 involved a review by an expert panel to determine the content and face validity indices of the instrument. Stage 3, as a pilot study, evaluated the dentistry evaluation program in the two departments of orthodontics and prosthodontics, Faculty of Dentistry, Tabriz University of Medical Sciences, based on reliable and valid researcher-made instruments.

### Step 1: designing the items

In this step, the questions were designed based on the CIPP model in the four domains. There were six items in the domain of context, six in input, six in process, and seven in output. The items were scored on a five-point Likert scale (*very low*, *low*, *moderate*, *high*, and *very high*). At the end of these items, some open-ended questions asked the participants to recommend methods for facilitating novel learner evaluation programs for the general dentistry program.

### Step 2: evaluating the reliability and validity of the instrument

To determine the validity of the questionnaire, its face and content validity was assessed upon a review by the expert panel. As there was no similar questionnaire for this purpose, criterion validity was not examined.

### Face validity

Face validity was assessed by two methods. In the quantitative method, to examine the content validity index (CVI), the method introduced by Waltz and Bausell was adopted, and the judgment of each member of the expert panel about each item was checked by three criteria of simplicity, clarity, and relevance on a four-point Likert scale. The minimum acceptable value for CVI, based on the expert panel size (n = 8), was 0.79, and if the CVI of an item was < 0.79, it was modified based on the experts’ opinion.^12^

To determine the content validity ratio (CVR), each member of the expert panel’s judgment about each item was evaluated on a three-point scale: 1 = *It is essential*, 2 = *It is useful but not essential*, 3 = *It is not essential*. Based on Lawshe’s table and the number of evaluators (n = 8), items whose CVR was > 0.75 were deemed essential.^13^

To examine face validity, the participants were asked to check the degree of importance of each item on a five-point Likert scale from 1 (*not important at all*) to five (*very important).* Only items with scores > 1.5 were deemed as having acceptable face validity.

The qualitative phase included a 90-minute focused group discussion with eight expert academicians, including two medical education experts and six dentistry faculty members. The feedback given on the items was discussed in the session, and some items were modified or deleted.

The reliability of the instrument was checked with Cronbach’s alpha. The finalized questionnaire was completed by 10 dentistry faculty members. Two weeks later, the same questionnaires were completed by the same group, and the internal consistency of the instrument was determined based on Cronbach’s alpha.

### Step 3: Piloting the questionnaire

The Departments of Orthodontics and Prosthodontics were selected for the pilot study. With the permission of the Dean of the Faculty, the questionnaires were completed by the faculty members of these departments and analyzed by descriptive statistics (mean and SD) in SPSS 19. Open-ended questions were analyzed by content analysis. In this analysis, sentences with semantic content were highlighted; their semantic content was extracted and coded, and similar codes were subsumed under categories.

## Results

The content and face validity of the tools were assessed by CVI, CVR, and the item impact score by seeking assistance from eight faculty members. The reliability of the tools was measured by Cronbach’s alpha. The content and face validity and the reliability of the program evaluation tool for the “implementation of novel clinical skills’ evaluation methods” from the viewpoint of dentistry faculty members based on the CIPP model were estimated and confirmed at CVR = 0.89, CVI = 0.92, and α = 0.76.

Twenty-five faculty members of the departments mentioned above completed the questionnaires. Table 1 presents the demographic data of the participants.

**Table 1:** The demographic data of the dentistry faculty academic staff members

**Department**	**Sex**	**Age**	**Academic work experience**	**Academic status**
Orthodontics	Male = 6 Female = 3	30-40 = 4 40-50 = 3 50-60 = 2	5-15 = 4 15-25 = 3 25-35 = 2	Assistant professor = 5 Associate professor = 4
Dental prosthesis	Male = 9 Female = 7	30-40 = 6 40-50 = 8 50-60 = 2	5-15 = 9 15-25 = 5 25-35 = 2	Assistant professor = 6 Associate professor = 10

The participants’ level of satisfaction with the context indices in the professional competency-based evaluation program was examined in two domains of “contextual factors related to the educational institution” and “contextual factors related to human resources.” The general level of satisfaction with the context indices was above average (55%). The highest satisfaction level belonged to the “compatibility of the department’s educational atmosphere for implementing novel evaluation methods” and “novel methods’ acceptance by and attraction for the students.” In contrast, the lowest satisfaction level belonged to the “physical compatibility of the department for administering the exams” (Table 2).

**Table 2:** Dentistry faculty academic staff members’ level of satisfaction with context indices in the professional competency-based evaluation program

**Domain**	**Item**	**Satisfaction level** **(Mean ± SD)** **N =25**
Contextual factors related to the educational institution	Sensing the provision of a support base by the university	53 ± 29.00
	Compatibility of the department’s educational atmosphere for implementing novel evaluation methods	64 ± 21.00
	Physical compatibility of the department for administering the exams	46 ± 25.00
Contextual factors related to human resources	The effect of administering novel exams on promoting the academic rank of faculty members	50 ± 24.01
	Faculty members’ motivation for using novel evaluation methods	55 ± 22.00
	Novel methods’ acceptance by and attraction for the students	60 ± 18.00
General satisfaction	The general level of satisfaction with context indices	55 ± 15.00

The participants’ satisfaction with input indices in the professional competency-based evaluation program was examined in three domains of “physical infrastructure of the faculty,” “human infrastructure,” and “professor empowerment.” The general satisfaction with input indices was below average (48%). The highest degree of satisfaction belonged to the “physical infrastructure of the faculty.” The participants had the highest level of satisfaction with the “quality of the designed evaluation instruments” and the “effect of content familiarity workshops to familiarize the faculty members with competency-based assessment methods,” while they had the lowest level of satisfaction with the “professors’ familiarity and mastery” (Table 3).

**Table 3:** The level of satisfaction with input indices in the professional competency-based evaluation program

**Domain**	**Item**	**Satisfaction level (Mean ± SD)** **N =25**
Physical infrastructure of the faculty	Compatibility of the departments’ educational facilities	48 ± 18.00
	Quality of the designed evaluation instruments	55 ± 22.00
Human infrastructure	Professors’ familiarity and mastery	44 ± 17.00
	Students’ familiarity and awareness	42 ± 11.00
Professor empowerment	Effect of content familiarity workshops to familiarize the faculty members with novel evaluation methods	51 ± 24.00
	Effect of workshops on the development and implementation of performance-based evaluation methods	50 ± 24.01
General satisfaction	The general level of satisfaction with input indices	48 ± 12.00

The participants’ level of satisfaction with the process indices of the evaluation program was examined in two domains of “design and implementation of performance-based evaluation methods” and “feedbacks on the implementation of performance-based evaluation programs.” The general level of satisfaction with process indices was above average (55%). The highest satisfaction level belonged to “cooperation between faculty members in implementing performance-based assessment methods” and “cooperation in designing clinical skills’ evaluation methods.” The lowest degree of satisfaction belonged to “receiving feedback from students to promote performance-based evaluation methods” (Table 4).

**Table 4:** Dentistry faculty academic staff members’ level of satisfaction with process indices in the professional competency-based evaluation program

**Domain**	**Item**	**Satisfaction level (Mean ± SD)** **N =25**
Design and implementation of performance -Based assessment methods	Cooperation between faculty members in designing clinical skills’ evaluation methods	67 ± 20.00
	Cooperation between faculty members in implementing competency-based assessment methods	69 ± 14.00
	Documented and wide-range implementation of performance-based evaluation methods in departments	57 ± 20.00
	Cooperation of faculty members in familiarizing the learners with performance-based evaluation methods	46 ± 27.00
Feedbacks on the implementation of performance-based evaluation methods	Amount of feedback received from faculty members to promote the performance-based evaluation methods	50 ± 24.01
	Amount of feedback received from students to promote the performance-based evaluation methods	44 ± 25.00
General satisfaction	The general level of satisfaction with input indices	55 ± 17.00

The participants’ satisfaction with the output of implementing performance-based evaluation methods was examined in three domains of “teaching and learning outcomes,” “promotion of professional and academic ethics,” and “implementation of the educational curriculum.” The general satisfaction level of faculty members with the outputs of performance-based evaluation methods was above average (58%). The highest degree of satisfaction belonged to “teaching and learning outcomes,” and more specifically, with “application of performance-based evaluation methods in teaching the practical and clinical aspects of the discipline” and “application of novel evaluation methods in forming learners’ learning experiences.” The lowest level of satisfaction belonged to the “application of performance-based evaluation methods in promoting professional ethics for learners” (Table 5).

**Table 5:** Dentistry faculty academic staff members’ level of satisfaction with output indices in the professional competency-based evaluation program

**Domain**	**Item**	**Satisfaction level (Mean ± SD)** **N =25**
Teaching and learning outcomes	Application of novel evaluation methods in teaching the practical and clinical aspects of the discipline	67 ± 22.00
	Application of novel evaluation methods in systematizing the teaching and learning process	58 ± 28.00
	Application of novel evaluation methods in forming learners’ learning experiences	60 ± 25.00
	Application of novel evaluation methods in standardizing and unifying the teaching and learning process	66 ± 20.00
Promotion of professional and academic ethics	Application of novel evaluation methods in promoting professional ethics for learners	44 ± 35.01
	Application of novel evaluation methods in ensuring educational equity	58 ± 31.00
Curriculum implementation	Application of novel evaluation methods for complete curriculum implementation	53 ± 25.00
General satisfaction	The general level of satisfaction with input indices	59 ± 23.00

**Figure 1:**
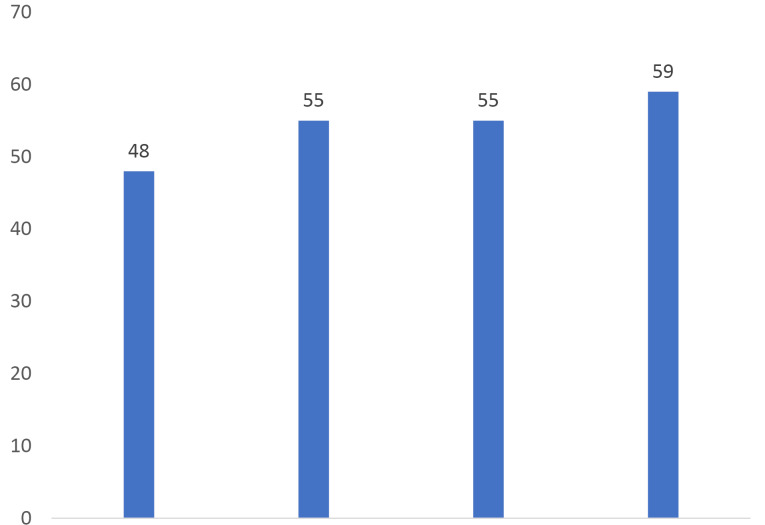
Faculty academic staff members’ general level of satisfaction with implementing the professional competency-based evaluation program based on CIPP indices.

The faculty members’ general satisfaction level was the highest with output indices and the lowest with the input indices (Figure 1).

The analysis of open-ended questions yielded two general categories of “providing the human and physical infrastructure” and “spiritual support and encouragement of educational innovation.”

In “providing the human and physical infrastructure,” two sub-categories of “dentistry professors’ empowerment” and “preparing physical facilities” were obtained.

### Dentistry professors’ empowerment

The empowerment of human resources is the driving force for changing the educational system. To bring about a paradigm shift from traditional educational methods to novel evaluation methods, human resources should be prepared for accepting such a change. The university should design professor empowerment courses based on the timetable. A participant from the Department of Prosthodontics said, “It is better to hold short but continuous and practical empowerment courses in the faculty. We don’t need theoretical concepts but workshops and practice. This is the best method for teaching busy learners.”

According to a participant from the Department of Orthodontics, “It’s better to hold a workshop for our discipline on designing checklists and writing scenarios. We attend most workshops with medical professors, where they become the center of attention, and we do not get to see tangible examples.”

### Preparing physical facilities

Preparing the inputs of program implementation is critical for program implementation. A change in assessment methods should be made in the faculty’s strategic plans, and its implementation requires a sufficient budget. “We do not have a test center to design standard stations or administer standard exams,” said a participant from the Department of Periodontics. According to a participant from the Department of Orthodontics, “For this number of students, we have few facilities and professors. We need many professors if we want to design Direct Observation of Procedurals Skills (DOPs) test for students, pay attention to the accurate implementation of procedures by the learners during the course, and ensure their authentic learning.”

In the category of “spiritual support and encouragement of educational innovation,” two sub-categories of “promotion of innovative professors” and “valuing educational innovations” were extracted.

### Promotion of innovative professors

The promotion system in medical sciences universities is mostly research-based, and educational innovations are not defined in the promotion system or have an insignificant role. Therefore, professors prefer to publish scientific articles in ISI-index journals and get promoted instead of innovating educational activities that are not reflected anywhere and do not lead to promotion.

A participant from the Department of Orthodontics said, “If we want innovation in education, we must change the promotion regulations. Educational and research faculty members are viewed as being the same in Iran. The university’s policy determines the professors’ performance.”

According to a participant from the Department of Periodontics, “The mission of the university should change towards innovation in education, and we should move in this direction. A change in our attitude towards education and evaluation needs a change in the university’s attitudes and regulations.”

### Valuing educational innovations

Educational innovations have not found their proper place in medical sciences universities, and professors come up with such innovations merely based on their personal interests. If the university modifies its values towards a student-centered approach and recruits innovative professors, it can motivate the professors to bring about changes and value the services of creative professors instead of regarding them as rebels who cause trouble for other professors.

A participant from the Department of Periodontics commented, “Other professors think novel evaluation and teaching methods are an extra burden, maybe because they receive no feedback from the Dean of the Faculty. If our feedback system is functional, positive feedback will serve as spiritual support, which encourages other professors to cooperate in the implementation of novel methods.”

According to a participant from the Department of Orthodontics, “Sometimes we don’t even receive written positive feedback, so we lose interest over time. The universities think a professor who gives only one final score to learners based on observing their performance over the semester is the same as one who designs DOPs forms and writes scenarios. That is why innovative professors become de-motivated.”

## Discussion

This study aimed to evaluate the “dentistry competency-based evaluation program” based on the CIPP model from dentistry faculty academic staff members’ viewpoint. The faculty members of periodontics and orthodontics departments exhibited moderate satisfaction with the professional competency-based evaluation program. To promote the quality of exams, the infrastructure should be prepared in all four domains. In terms of context, the faculty members believed that the university does not provide a supportive context for the implementation of performance-based evaluation methods, and the physical facilities of the department are not suitable. Still, the atmosphere of the department and the novel methods’ acceptance by and attraction for the students were better compared to the other items. Therefore, faculty members and students are ready to accept novel evaluation methods. In terms of input, faculty members held that the educational facilities of the department are not appropriate. They also declared in open-ended questions that the department does not have an examination room or facilities for administering such tests. Similarly, in a study by Rezapour Mirsaleh et al,^14^ professors and students at Ardakan University (Iran) believed that the resources and equipment were not optimal. Based on studies on dental schools in Tehran and Babol University of Medical Sciences, the equipment of periodontics and oral health departments was insufficient.^8,15^ Of course, the fact that these evaluation methods are new might explain the lack of sufficient equipment for administering these exams. Also, professors have not yet mastered the design and implementation of such exams. The participants mentioned in open-ended questions that there is no separate professor empowerment course for dentistry; therefore, they do not become familiar with objective scenarios appropriate for their discipline and test design techniques in the existing educational workshops. They believed that it would be better to hold workshops in the Faculty of Dentistry and between the class times so that they could participate in them. In the study by Makarem et al,^9^ a major problem in educational inputs of oral health and social dentistry was professors’ insufficient skills and education. Another problem was the students’ lack of awareness. The use of novel evaluation methods without raising the learners’ awareness leads to stress and confusion in their studies.^16^ Compared to written exams and multiple-choice tests, novel evaluation methods are stressful for students. According to Zartman et al,^17^ in dentistry, performance examination increases learners’ hand tremors and changes their voice; in some cases, these have prevented the learner from taking the exam. In a study by Hassell^18^ on rheumatology, the OSCE (Objective structured clinical examination) exam caused excessive stress in learners.Unfamiliarity with these exams can exacerbate this stress. Various studies have shown that the expression of expectations from the learners and the evaluation method at the beginning of each course directs the learners’ method of learning and studying.^19^ In the study by Makarem et al, vague objectives and expectations from learners were a weakness of educational input.^9^ In the domain of process, the highest satisfaction level was with the cooperation between faculty members in designing and implementing the exams.

The professors are ready for a paradigm shift from traditional to novel evaluation methods, but they need support and encouragement from the faculty. Professors become de-motivated because they do not receive feedback from the faculty and students. They mentioned in open-ended questions that they do not receive any positive feedback from the faculty, and their innovation does not affect their promotion; the promotion regulation is research-based and does not pay enough attention to educational innovations.^20^

According to the professors, novel evaluation methods help the learners’ practical learning and the formation of their learning experiences. These exams aim to ensure the learners’ mastery of the capabilities required by the course.^21^ However, the professors believed that the implementation of these exams was weak in terms of the complete curriculum implementation as it does not provide enough facilities for designing exams based on the capabilities required by the course. Moreover, the professor-to-student ratio is low, and professors cannot evaluate all the capabilities with these methods. In the qualitative study, by examining the educational problems of the school of dentistry, Qazvin University of Medical Sciences, a major challenge was the low professor-to-student ratio in most departments.^22^ Another reason for the poor performance of these exams in implementing the curriculum of dentistry could be that these exams are new, and professors are still in the first stages of exam design and adaptation to ensure the acquisition of capabilities. The overall results of the evaluation revealed that preparing the infrastructure, internalizing the use of novel evaluation methods, and supporting and encouraging the professors by giving them educational incentives and promotion can help implement these exams.

## Conclusion

The results indicated that, according to the faculty academic staff members, the implementation of competence-based evaluation methods in two departments of Periodontics and Orthodontics has infrastructural problems and is far from the standard administration of these exams. Some modifications, such as preparing the standard setting and facilities for administering the exams, empowering the professors, and defining the status of these exams in the curriculum and their role in ensuring the learners’ capabilities can enhance the quality of practical exams to approach the standards of advanced countries.

## Authors’ Contributions

RN, HH, SF, and NB prepared the manuscript. NB was responsible for coordinating the study. RN and SF distributed the questionnaires, coordinated FGDs, and interviewed the participants. RN and HH commented on the coding process and analyzed the questionnaires. HH wrote the first draft of the paper. RN, SF, and NB reviewed the first draft of the paper and improved it. All authors have read and approved the final version of the manuscript.

## Acknowledgments

The authors would like to thank Tabriz University of Medical Sciences for supporting and granting this research as a part of an MS thesis in medical education and the dental faculty members who participated in this study.

## Funding

This research was supported and granted by the Medical Education Research Center of Tabriz University of Medical Sciences, Tabriz, Iran. The funder had no responsibility for the design of the study, data collection, analysis, and writing of the manuscript.

## Competing Interests

No competing interests.

## Ethics Approval

This study was approved by the Research Ethics Committee of Tabriz University of Medical Sciences (IR.TBZMED.REC.1397.254). Data collection was carried out after obtaining verbal and signed informed consent from the participants following the Deceleration of Helsinki. The informed consent process provided participants the opportunity to ask questions and consider all options. Participants could refuse to participate or withdraw from the study at any time.
